# Tachykinergic signaling mediated by NK1 receptors in the respiratory parafacial region drives respiratory output and contributes to the chemoreflex response to CO_2_

**DOI:** 10.1007/s00424-025-03147-8

**Published:** 2026-01-23

**Authors:** Octávio A. C. Maia, Luiz M. Oliveira, Jan-Marino Ramirez, Ana C. Takakura, Thiago S. Moreira

**Affiliations:** 1 Department of Physiology and Biophysics Institute of Biomedical Sciences, University of São Paulo, Av. Prof. Lineu Prestes, 1524, São Paulo, 05508-000 SP Brazil; 2 Center for Integrative Brain Research, Seattle Children Research Institute, Seattle, WA 98101 USA; 3 Department of Neurological Surgery, University of Washington, Seattle, WA 98101 USA; 4 Department of Pharmacology, Institute of Biomedical Science, University of Sao Paulo, Sao Paulo, 05508 Brazil

**Keywords:** Parafacial region, Tachykinergic signaling, Breathing, Substance p, Central chemoreflex

## Abstract

The respiratory parafacial region (pFRG) contributes critically to central chemoreception, CO_2_/H^+^ homeostasis and the regulation of all major components of the respiratory rhythm. The respiratory rhythm is also modulated by the neuropeptide substance P which is released by tachykinin-1 (Tac1)- expressing neurons. However, how tachykinergic signaling modulates the pFRG region remains incompletely understood. Here we show that substance P (1 µM, 30 nL) microinjected into the ventral pFRG increased respiratory frequency, amplitude, and minute volume under baseline conditions. These excitatory effects were abolished by prior application of the NK1 receptor antagonist GR82334, confirming NK1-mediated effect. Bilateral blockade of NK1 receptors in the ventral pFRG significantly attenuated hypercapnia-induced (FiCO_2_= 0.07) increases in respiratory parameters, suggesting tachykinergic signaling in CO_2_ chemoreflex responses. Immunohistochemical analysis revealed a predominant co-localization of NK1 receptors with VGlut2-expressing glutamatergic neurons in the ventral pFRG, whereas minimal co-expression was found with GABAergic neurons. To directly assess the function of tachykinin-producing neurons, we optogenetically stimulated Tac1-expressing neurons in the ventral pFRG. Light activation produced immediate increases in respiratory rate and motor output. These findings demonstrate that tachykinin signaling in the ventral pFRG enhances respiratory output. Tac1-expressing neurons may function as an integrative hub for coordinating respiratory functions under baseline and chemoreflex conditions.

## Introduction

Breathing is a vital rhythmic behavior tightly regulated by a distributed network of brainstem circuits that integrate sensory information and modulate motor output to maintain homeostasis. Central chemoreception, the process by which the nervous system detects changes in arterial CO_2_/H⁺ levels, plays a crucial regulatory role. Among the brainstem regions implicated in chemoreception, the parafacial respiratory group (pFRG) has emerged as a key site for rhythm modulation and CO_2_ sensitivity, particularly in neonates and during states of heightened respiratory demand [11, 17, 34, 36, 42].

The parafacial respiratory group (pFRG) and the retrotrapezoid nucleus (RTN) refer to distinct, though partially overlapping, components of the ventral lateral medulla. The RTN is best defined as a functional population of Phox2b and neuromedin B-expressing, glutamatergic neurons that mediate central chemoreception by responding to changes in CO₂/H⁺ [26, 52, 56]. In contrast, the pFRG encompasses a broader anatomical region on the ventral medullary surface, rostral and ventral to the facial nucleus, that contains multiple neuronal subpopulations involved in respiratory patterning [22, 23, 50, 58]. These include not only the RTN chemosensitive domain but also neurons implicated in inspiratory modulation and, more controversially, putative expiratory-related neurons [22, 30, 45]. Thus, while the RTN represents a well-defined chemoreceptive phenotype, the pFRG denotes a heterogeneous anatomical territory with diverse respiratory functions. These neurons are known to express neurokinin 1 (NK1) receptors, which bind substance P (SP), a neuropeptide encoded by the Tac1 gene [6, 15, 40, 66]. NK1 receptor (NK1-R) containing neurons are broadly distributed along the ventral respiratory column (VRC) [2], yet the only region of the VRC that has been studied extensively is the pre-Bötzinger complex (preBötC). Indeed, the presence of NK1-R in the preBötC has long been used as a marker for neurons critical for inspiratory rhythmogenesis [4, 15, 21, 44, 46, 48]. By contrast, the functional roles of tachykinergic signaling in the pFRG/RTN remain poorly understood, especially under normal physiological conditions or during respiratory challenges such as hypercapnia.

Pharmacological, genetic, and neurophysiological studies have shown that SP can enhance respiratory output, and NK1-R antagonists or genetic ablation of neurokinin neurons and their underlying cellular mechanisms attenuate respiratory responses [6, 12, 31, 55, 61, 66], but these effects have not been fully mapped within the pFRG/RTN. Moreover, whether neurons that produce SP actively contribute to breathing and autonomic control in vivo has yet to be determined. This gap in knowledge is especially relevant given recent evidence that pFRG/RTN neurons participate in respiratory-sympathetic coupling, a mechanism critical for coordinating breathing and cardiovascular function [33, 57].

In the present study, we investigated the role of tachykinergic signaling in the ventral pFRG (which includes the RTN) in modulating respiratory activity under baseline and hypercapnic conditions. We first tested whether microinjection of SP into the pFRG/RTN region could modulate breathing and whether this effect was mediated by NK1-R. We then examined the contribution of NK1-R to the hypercapnic ventilatory response using targeted pharmacological blockade. To better understand the cellular identity of neurons involved in this pathway, we characterized the co-expression of NK1-R with glutamatergic and GABAergic markers. Finally, using optogenetic activation of Tac1 gene-expressing neurons, we assessed the impact of these peptidergic cells on respiratory outputs in anesthetized mice. Together, these experiments provide new insights into the functional role of substance P sensitive neurons in the pFRG/RTN, revealing their involvement in the respiratory modulation.

## Material and methods

### Ethical approval

All experiments and surgical procedures were performed in accordance with the guidelines set by National Institutes of Health (NIH Publications Nº 8023, revised 1978) and the Institutional Animal Care of the University of São Paulo, and were approved by the Institutional Animal Care and Use Committee at the University of São Paulo (ID: CEUA-ICB/USP 2781260620) and the Seattle Children’s Research Institute Institutional Animal Care and Use Committee (ID: IACUC00058). All efforts were made to minimize pain and suffering. The investigators acknowledge the ethical principles upheld by the journal and confirm that the present work complies with the animal ethics checklist.

### Animals

Mice were housed in ventilated racks and steam-sterilized caging (up to 4 per cage), with *ad libitum* access to food and water. Animals were maintained on a 12 h light/dark cycle in a vivarium maintained at 22–24 °C and ~ 40–50% relative humidity. To characterize neuroanatomical patterns, we crossed VGlut2^Cre/+^ mice (Slc17a6^tm2(cre)Lowl^/J; JAX#016963), or VGat^Cre/+^ mice (Slc32a1^tm2(cre)Lowl^/J; JAX#016962) with a Cre-dependent reporter line (B6.Cg-Gt(ROSA)26Sor^tm6(CAG−ZsGreen1)Hze^/J; Ai6; JAX#007906). The offspring from these crosses are referred to as VGlut2^Cre/+^::Ai6 and VGat^Cre/+^::Ai6 mice, respectively.

For optogenetic experiments, we crossed Tac1^Cre/+^ mice (Tac1^tm1.1(cre)Hze^; JAX#021877) with a Cre-dependent reporter line (B6.Cg-Gt(ROSA)26Sor^tm6(CAG−ZsGreen1)Hze^/J; Ai6; JAX#007906) [18]. The offspring from this cross is referred to as Tac^Cre/+^::Ai6. For pharmacology experiments, we used C57BL/6J mice (JAX#000664) or the Chat^Cre/+^::Ai6 (B6.129 S-Chat^tm1(cre)Lowl^/MwarJ; JAX#031661). A total of 45 mice of both sexes were used for anatomical and physiological assessments.

### Viral vectors

For optogenetic stimulation of pFRG/RTN Tac1-expressing cells, we used AAV1-EF1a-double-floxed-hChR2(H134R)-mCherry-WPRE-HGHpA (Addgene plasmid 20297, titer for injection: 12.1 × 10^13^ GC/ml).

## Pharmacological and optogenetic experiments in vivo

### Physiological preparation

General anesthesia was induced in mice with 5% isoflurane delivered in 100% O_2_. The isoflurane concentration was reduced to 1.4–1.5% until the end of the surgical process. All mice (C57BL/6J, ChAT^Cre/+^ or Tac^Cre/+^) used for anesthetized experiments were subjected to the following surgical procedures: (1) Placed in dorsal (pharmacology experiments) or supine (optogenetic experiments) position in the stereotaxic apparatus (model Kopf 1760) and the trachea was exposed through a midline incision and cannulated caudal to the larynx with a curved (180°) tracheal tube (PTFE 24G, Component Supply, Sparta, TN); (2) Removal of the occipital bone for the insertion of a pipette for drug injection or a fiber optic for laser stimulation directly into the ventral aspect of the pFRG region; (3) Implantation of electrodes for external intercostal muscle (Int_EMG_) or diaphragm muscle (Dia_EMG_) recording. For Int_EMG_ or Dia_EMG_ two thin teflon-coated silver wires with bared tips forming a 2 mm hook were inserted through the lateral edge of the intercostal on the right side of the mice or by placing the electrodes in the costal diaphragm. The electrode tips were inserted no more than 2–3 mm apart to minimize the electrocardiogram (EKG) artifact; (4) Isolation of the cervical vagus (cVN) and the hypoglossal nerve (XII). All the recorded nerves were isolated unilaterally, cut distally, and recorded via a unipolar suction electrode connected to a fire-polished, pulled glass pipette filled with aCSF. The corresponding contralateral nerve was preserved and remained intact. The cVN and the XII nerves were isolated, and the trachea and esophagus were cut and detached at the rostral end.

Upon completion of the surgical procedures, isoflurane was replaced by urethane (1.4 g/kg, intraperitoneal - i.p.) administered slowly. The adequacy of anesthesia was monitored during a 20-min stabilization period by testing for the absence of withdrawal responses, changes in Int_EMG_ or Dia_EMG_ to a firm toe pinch. Approximately hourly supplements of one-third of the initial dose of urethane were administered as needed to maintain an adequate level of anesthesia. Int_EMG_ or Dia_EMG_, cVN and XII signals were digitized with a micro1401 (Cambridge Electronic Design), stored on a computer, and processed offline using Spike2 software (version 7.2, Cambridge Electronic Design, Cambridge, UK). Integrated intercostal or diaphragm muscle activity (∫EMG) and integrated nerves were obtained after rectification and smoothing (τ = 0.015 s) of the original signal, which was acquired with a 30–300 Hz bandpass filter. The activity of respiratory muscles and nerves was quantified based on peak amplitude (in volts) and inspiratory burst frequency (by measuring the time interval over 20 consecutive bursts - expressed as breaths per minute), as observed in the integrated recordings of the intercostal or diaphragm muscles, the cervical vagus, and hypoglossal nerves. Neural minute × volume (Int_EMG_ fr × ampl, a measure of the total muscle activity per unit of time) was determined by averaging Int_EMG_ during 20 respiratory cycles and normalizing the result by assigning a value of 0 to the dependent variable recorded at low levels of end-expiratory CO_2_ (below threshold) and a value of 1 at the highest level of P_CO2_ investigated (between 9.5 and 10%).

EMGs and nerves amplitude and frequency were evaluated before and after pharmacological administration and during photostimulation. To obtain control values, the 10 s preceding each experimental manipulation for all parameters were averaged. As the time of the peak response to drug injections was different on each channel, for values for the effect of drug injections, 10 s were averaged at the peak of change on each channel separately.

### Photostimulation and pharmacological injection in the ventral aspect of the respiratory parafacial region

The light source was a diode pumped 473 nm blue laser (Thorlabs laser Model S1FC473MM; Newton, NJ, USA) controlled by a function generator (Grass Technologies/Astro-Med Inc., Warwick, RI, USA) to generate 10 ms light pulses. Stimulation trials generally consisted of 100 s trains for 10 ms pulses delivered at 15 Hz. Five photostimulation trials were conducted for each animal, and the data presented represent average values from these trials. Trials were included in the analysis if they met predefined criteria: stable baseline breathing before stimulation and a clear response during stimulation, minimizing variability due to factors like movement artifacts or irregular baseline states. The actual power output measured at the end of the fiber with a light meter (Thorlabs, Newton, NJ, USA) was close to 9 mW. The same fiber optic was used for all experiments. Photostimulations were made by placing the fiber optic unilaterally in the ventral surface of the brainstem overtop of the predetermined pFRG/RTN region.

Uni- or bilateral injections of substance P (1 µM in sterile saline − 30 nL, pH 7.4, Sigma Chemicals Co), GR82334 (selective NK1-R antagonist, 100 mM in sterile saline − 30 nL, pH 7.4, Sigma Chemicals Co) or saline into the ventral pFRG region were performed using nitrogen pressure (8–15 ms pulses) via glass micropipettes (0.5 mm inner diameter, Sutter Instrument Co, CA) coupled to a PicoSpritzer II pneumatic pump (General Valve Corporation, Fairfield, NJ, USA). All drug concentrations were selected based on previous studies on breathing regulation [7, 9, 38, 39, 65]. The injection volume was maintained at approximately 30 nL, as indicated by graduated rule coupled with scope used to observe the surface of animal’s head during the surgery. In addition, substance P, GR82334 or saline contained 1% fluorescent latex microspheres (Lumafluor, New York City, NY, USA) for subsequent histological analysis. Injections were made using the following coordinates to target the pFRG/RTN of mice: 5.5 mm below the dorsal surface of the brain, 1.3 mm lateral to the midline, and 1.4 mm caudal to lambda.

Following the completion of pharmacological or optogenetic stimulation, the depth of anesthesia was carefully verified by an absence of the hind-paw withdrawal reflex to ensure animals were adequately anesthetized. The animals were then immediately perfusion-fixed for subsequent histological analysis as described below.

#### Hypercapnia ventilatory response

The ventilatory response to hypercapnia was assessed by exposing the mice to 7% inspired CO_2_ (FiCO_2_ = 0.07) for 2 min under hyperoxic conditions (FiO_2_ = 0.93). Each animal underwent four hypercapnia sessions: The first and second epochs were recorded 10 and 20 min after bilateral microinjections of saline, while the third and fourth epochs were recorded 10 and 20 min after injections of GR82334 into the ventral aspect of the pFRG region.

#### Histology, analysis, and cell count

Immunohistochemical procedures were performed as previously described [32, 35, 51]. Mice were first deeply anesthetized with ketamine/xylazine (100/10 mg/kg, i.p.). After confirming the absence of the hind-paw withdrawal reflex, 50 units of heparin were injected transcardially. The mice were then perfused via the ascending aorta and pulmonary artery with 50 ml of PBS (pH 7.4), followed by 100 ml of 4% formaldehyde (Electron Microscopy Sciences, Fort Washington, PA, USA) in 0.1 M phosphate buffer (PB, pH 7.4). The brains were then removed and stored in fixative for 24–48 h at 4 °C. A series of coronal Sect. (1:4 series, 30 μm thick) were cut along the rostrocaudal axis using a microtome (SM2010R; Leica Biosystems, Buffalo Grove, IL Vibratome) and stored at −20 °C in cryoprotectant solution (20% glycerol, 30% ethylene glycol in 50 ml PB) for later histological processing. All histochemical procedures were performed using free-floating sections, in accordance with previously described protocols [32, 35, 51].

For the immunofluorescence technique, NK1-R was detected using a polyclonal rabbit anti-NK1 antibody (1:2000; Sigma-Aldrich, Saint Louis, MO, USA); tyrosine hydroxylase (TH) was detected using a polyclonal mouse anti-TH antibody (MAB 318; Millipore; 1:1000); Phox2b was detected using a polyclonal rabbit anti-Phox2b antibody (AB 2813765, Santa Cruz Biotechnology; 1:100); mCherry protein was detected using a polyclonal rabbit anti-DsRed antibody (Takakara 632496; 1:1000); and GFP was detected using a polyclonal chicken-anti-GFP (AB 13970, Abcam; 1:10,000). Each sample was diluted in PBS containing normal donkey serum (017-000-121; 1%; Jackson Immuno Research Laboratories) and 0.3% triton and then incubated for 24 h. The sections were subsequently washed in PBS and incubated for 2 h with donkey anti-mouse DyLight 405 (715-475-150; Jackson Immuno Research Laboratories; dilution 1:100); donkey anti-rabbit Alexa 647 (715-605-152; Jackson Immuno Research Laboratories; dilution 1:400); donkey anti-rabbit Alexa 594 (711-585-152; Jackson Immuno Research Laboratories; dilution 1:400) or donkey anti-chicken Alexa 488 (703-545-155; Jackson Immuno Research Laboratories; dilution 1:400).

The brain sections were mounted onto slides, dried, covered with DPX (Aldrich, Milwaukee, WI, USA), and coverslips were affixed with nail polish. Brain sections were analyzed under blind conditions using StereoInvestigator software (MBF Bioscience) with a confocal fluorescence microscope with High Content Imaging in Cell Analyzer 2200 GE (Zeiss LSM 780-NLO, Oberkochen, Germany). Images were acquired with a Hamamatsu C11440 Orca-Flash 4.0LT digital camera (resolution: 2048 × 2048 pixels) resulting in TIFF files. A technical illustration software package (Adobe Illustrator, v. 28.1, USA) was used for line drawings, figure assembly, and labeling, following the guidelines of Paxinos & Franklin [13].

The sections were counted bilaterally, and the numbers reported in the results section correspond exactly to the counts of one-in-four sections in a series. Section alignment between brains was completed relative to a reference section, as previously described [35, 41]. Briefly, to align sections around the pFRG/RTN level, the most caudal section that contained an identifiable cluster of facial motor neurons was identified in each brain and assigned to the level of 6.48 mm caudal to the bregma (Bregma level = − 6.48 mm). Rostral or caudal levels to this reference section were determined by adding or subtracting the number of intervening Sect. (30 μm intervals). The analysis was performed as follows: 1) pFRG/RTN: 5 sections rostral from the caudal end of the facial nucleus (bregma: −6.00 to −6.48 mm). For the pFRG/RTN level, the mapping was limited to the ventral half of the brainstem which contains the distinctive and isolated parafacial cluster of Phox2b^+^-expressing neurons [35, 52].

#### Statistical analysis

All data used in inferential statistical tests were assessed for normality using the Shapiro-Wilk test (based on sample size) and confirmed to follow a normal distribution. For normally distributed data, the Student´s t test, one- or two-way repeated measures ANOVA, followed by Bonferroni´s multiple comparisons test was used. Paired t-tests were used for within-subject comparison. Paired t-test was used to examine: (1) hypercapnia ventilatory response (7% CO_2_) under control condition (saline injection into the pFRG), and in the presence of NK1-R antagonist GR82334 bilaterally injected into the pFRG; (2) differences in Dia_EMG_, cVN and XII activities following optogenetic stimulation of Tac-1 neurons in the pFRG region. The F- and p-values for every effect and interaction between effects are reported in the figure legends. Results are shown as mean ± SD and individual points. Statistical analyses were performed using GraphPad Prism (v.9).

## Results

### Substance P injection in the ventral respiratory parafacial region increases respiratory activity

**Fig. 1:**
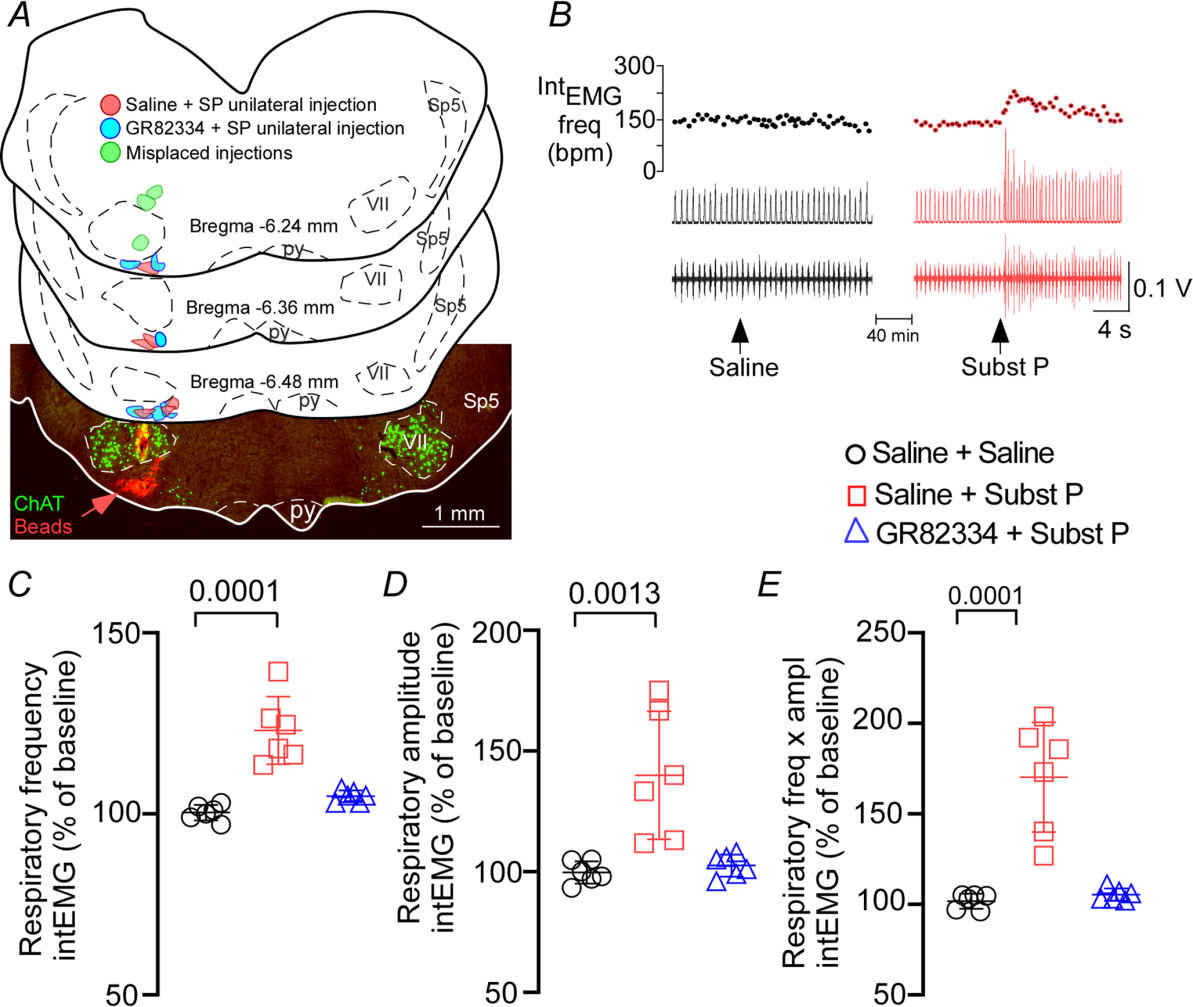
Substance P in the ventral aspect of the respiratory parafacial region increases breathing in anesthetized mice. (**A**) Computer-generated plot of unilateral injections of saline + substance P (red; *N* = 6), NK1 antagonist - GR82334 + substance P (blue; *N* = 6) or misplaced injections (green; *N* = 3) into the pFRG/RTN region in the Chat^Cre/+^::Ai6 mouse (coronal projection on plane bregma − 6.24 to −6.48 mm of the atlas by Paxinos & Franklin) [13]. The photomicrography shows a representative unilateral injection (red beads) into the pFRG/RTN region in the Chat^Cre/+^::Ai6 mouse. (**B**) Intercostal electromyography (Int_EMG_) frequency (upper panel), integrated (middle panel), and raw (lower panel) recordings showing the effect of unilateral injection of saline (control) or substance P (1 µM − 30 nL) into the pFRG/RTN region. Percentage increase of baseline of (**C**) Int_EMG_ frequency (F_2,15_ = 27.42; *p* < 0.0001), (**D**) Int_EMG_ amplitude (F_2,15_ = 12.24; *p* < 0.0007), and (**E**) Int_EMG_ minute volume of the activity recorded after unilateral injections of saline or substance P into the pFRG region, before and after administration of the NK1 receptor antagonist GR82334 (100 nM, 30 nL) (F_2,15_ = 28.05; *p* < 0.0001). *N* = 6/group of mice. Abbreviations: py, pyramidal tract; Sp5, spinal trigeminal tract; VII, facial motor nucleus. Scale in A = 1 mm

 Figure 1A illustrates the typical site of unilateral injections in the ventral aspect of the respiratory parafacial region. The injection center was located approximately 100 μm below the facial motor nucleus and 100–150 μm rostral to the caudal portion of the facial nerve motor nucleus, lateral to the pyramidal tract, and medial to the spinal trigeminal tract. The latex microspheres (marked in red) were used to mark the sites of injection and to evaluate the dispersion of approximately 50–100 μm in the rostrocaudal direction from the center of the injection (data not shown). It is important to notice that injections located outside the pFRG/RTN region, i.e. injections centered in the facial motor nucleus (*N* = 1) or in the dorsal region of the facial motor nucleus (*N* = 2) did not produce changes in breathing activity (t = 0.257; *p* > 0.05) (data not shown). Unilateral injection of substance P (SP: 1 µM − 30 nL) in the ventral pFRG region, under baseline conditions, exerted an excitatory effect on the respiratory system as demonstrated in the representative electromyographic tracing of the external intercostal muscle (Fig. 1B). For example, we observed a significant increase in respiratory rate (Int_EMG_ frequency) (123 ± 9.4 vs. saline: 100 ± 2.2% of baseline) (F_2,15_ = 27.42; *p* < 0.0001), Int_EMG_ amplitude (140 ± 26.5 vs. saline: 99.7 ± 4.6% of baseline) (F_2,15_ = 12.24; *p* = 0.0007) and Int_EMG_ minute volume (170.3 ± 30.4 vs. saline: 101.6 ± 4.2% of baseline) (F_2,15_ = 28.05; *p* < 0.0001) (Figs. 1 C-E). The increase in respiratory activity induced by SP injection into the ventral pFRG was entirely mediated by NK1 receptors. This was demonstrated by the fact that prior injection of the NK1 receptor antagonist GR82334 (100 mM, 30 nL) completely blocked the SP-induced increases in Int_EMG_ frequency (104.8 ± 1.6% vs. saline: 100 ± 2.2% of baseline; *p* = 0.3726), Int_EMG_ amplitude (102.5 ± 4.6% vs. saline: 99.7 ± 4.6% of baseline; *p* = 0.9486), and Int_EMG_ minute volume (105.3 ± 3.4% vs. saline: 101.6 ± 4.2% of baseline; *p* = 0.9372) (Figs. 1 C-E).

### Blockade of the tackykinin1 receptors in the ventral respiratory parafacial region reduces hypercapnia-induced increase in respiratory activity

**Fig. 2:**
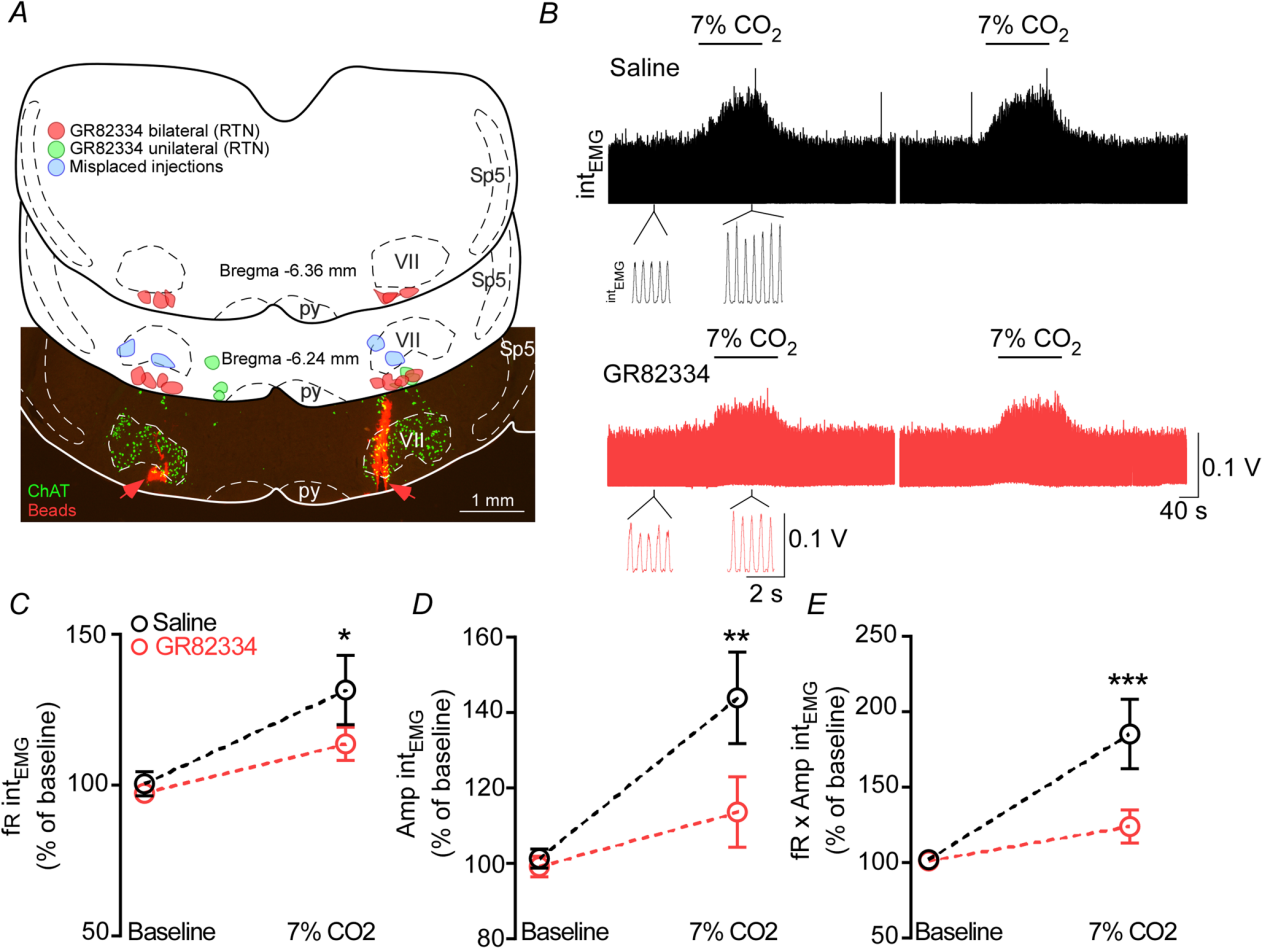
Respiratory effects elicited by blockade of the tachykinergic receptors in the respiratory parafacial region on the hypercapnic ventilatory response. (**A**) Computer-generated plot of unilateral (green; *N* = 3), bilateral (red; *N* = 7) or misplaced (blue; *N* = 2) injections into the pFRG/RTN region in the Chat^Cre/+^::Ai6 mouse (coronal projection on plane bregma − 6.24 to −6.36 mm of the atlas by Paxinos & Franklin). The photomicrography shows a representative bilateral injection (red beads) into the pFRG/RTN region in the Chat^Cre/+^::Ai6 mouse. (**B**) Integrated intercostal electromyography (Int_EMG_) recording showing the effect of hypercapnia (7% CO_2_) after the bilateral injection of saline (control) or the NK1 antagonist (GR82334–100 mM − 30 nL) into the pFRG/RTN region. Percentage changes of 7% CO_2_ elicited by bilateral injection of saline (control) or GR82334 (100 mM − 30 nL) into the pFRG/RTN under hypercapnia (7% CO_2_) challenge in (**C**) Int_EMG_ frequency. Two-way ANOVA repeated measures, baseline vs. 7% CO_2_ (F_1,12_ = 82.38; *p* < 0.0001); saline vs. GR82334 (F_1,12_ = 17.96; *p* = 0.0012), and interaction (F_1,12_ = 7.88; *p* = 0.0158), (**D**) Int_EMG_ amplitude. Two-way ANOVA repeated measures, baseline vs. 7% CO_2_ (F_1,12_ = 128.7; *p* < 0.0001); saline vs. GR82334 (F_1,12_ = 22.95; *p* = 0.0004), and interaction (F_1,12_ = 31.25; *p* = 0.0001), (**E**) Int_EMG_ minute volume. Two-way ANOVA repeated measures, baseline vs. 7% CO_2_ (F_1,12_ = 128.8; *p* < 0.0001); saline vs. GR82334 (F_1,12_ = 38; *p* < 0.0001), and interaction (F_1,12_ = 41.67; *p* < 0.0001). *N* = 7/group of mice. Abbreviations: py, pyramid; Sp5, spinal trigeminal tract; VII, facial motor nucleus. Scale bar in A = 1 mm

 As expected, hypercapnia (Fi_CO2_ = 0.07) elicited immediate changes in respiratory activity in animals that received bilateral injections of saline (Fig. 2B). The rapid increase in CO_2_ levels raised the Int_EMG_ frequency (100 ± 3.9 vs. 7% CO_2_: 131 ± 11.6% of baseline; F_1,12_ = 82.38; *p* < 0.0001), the Int_EMG_ amplitude (101.3 ± 2.6 vs. 7% CO_2_: 143.9 ± 12.15% of baseline; F_1,12_ = 128.7; *p* < 0.0001), and the Int_EMG_ minute volume (101.9 ± 2.5 vs. 7% CO_2_: 185.2 ± 23% of baseline; F_1,12_ = 128.8; *p* < 0.0001) (Figs. 2B-E). Bilateral blockade of NK1 receptors in the ventral pFRG using the NK1 receptor antagonist GR82334 (100 mM, 30 nL) significantly reduced the hypercapnia-induced increases in Int_EMG_ frequency (131 ± 11.6 vs. 7% CO_2_ under GR82334 : 113.7 ± 5.46% of baseline; F_1,12_ = 17.96; *p* = 0.0012), Int_EMG_ amplitude (143.9 ± 12.15 vs. 7% CO_2_ under GR82334: 113.6 ± 9.34% of baseline; F_1,12_ = 22.95; *p* = 0.0004), and IntE_MG_ minute volume (185.2 ± 23 vs. 7% CO_2_ under GR82334: 123.9 ± 10.9% of baseline; F_1,12_ = 38; *p* < 0.0001) (Figs. 2B-E). In the GR82334 group, Int_EMG_ frequency (*p* = 0.784), Int_EMG_ amplitude (*p* = 0.852) and Int_EMG_ minute volume (*p* = 0.99) were similar to control rats (Figs. 2B-E) As previously described, bilateral injections of GR82334 were placed in the portion containing the large population of chemosensitive neurons in the pFRG/RTN region (Fig. 2 A). The schematic figure illustrates injection sites of GR82334 centered in the pFRG/RTN or adjacent areas, including placements within the facial motor nucleus (*N* = 2). Unilateral blockade of tachykinin receptors (*N* = 3) or injections outside the pFRG region (*N* = 2) did not alter the respiratory responses to hypercapnia (data not shown).

### Co-localization of glutamatergic neurons in the ventral respiratory parafacial region with NK1 receptors

**Fig. 3:**
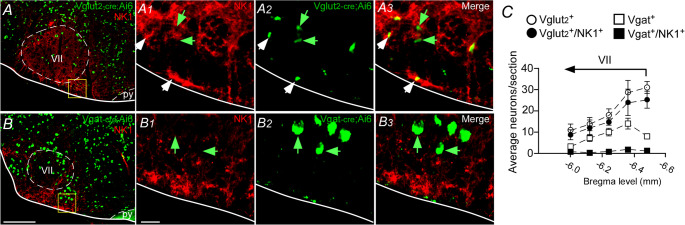
Excitatory colocalization with tachykinergic neuronal profile in the respiratory parafacial region. Photomicrographs of the ventral aspect of the pFRG/RTN region showing immunohistochemistry for NK1 receptors (Alexa 647, red) in (**A**) VGlut2^cre/+^::Ai6 mice (green) or (**B**) VGat^cre/+^::Ai6 (green). A1-3 and B1-3) Enlarge photomicrograph of the ventral medullary surface from figures A and B, showing the double label neurons in the pFRG region. (**C**) Average neurons/section detected in 5 sections throughout the bregma levels in the pFRG/RTN region (VGlut2^+^, all VGlut2-ir neurons irrespective of other markers; VGat^+^, all VGat-ir neurons irrespective of other markers). *N* = 4/group of mice). Scale bar = 100 μm in B applied to A; 20 μm for panel B1 applied to A1-A3 and B1-B3

 Immunoreactive terminals for the NK1-R are abundant in the ventral aspect of the brainstem [6, 40, 60]. To confirm that NK1-R of the ventral aspect of the pFRG region co-localize with glutamatergic (VGlut_2_) or GABAergic (VGat) neurons, we used the VGlut_2_^Cre/+^::Ai6 or VGat^Cre/+^::Ai6 reporter mouse lines along with NK1-R immunohistochemistry to identify the neuronal profile (Figs. 3A-B). Our analysis focuses on the ventral aspect of the pFRG region (Bregma level: −6.48 to −6.0 m; 1 series of 30 μm-tick coronal sections: 120 μm apart). As illustrated in Fig. 3 A, we found that the majority of the VGlut_2_-expressing neurons in the ventral aspect of the pFRG region colocalize or have presumably synapse contact with NK1 receptors (92.5 ± 9.4 vs. VGlut_2_^+^: 112 ± 7.9 neurons) (Fig. 3 C). In contrast, our analysis showed very few co-localizations between GABAergic neurons with NK1-R expression (4.75 ± 0.5 vs. VGat^+^: 44.25 ± 5.5 neurons) (Fig. 3 C).

### Optogenetic stimulation of Tac1-expressing cells into the ventral parafacial region in anesthetized mice

**Fig. 4:**
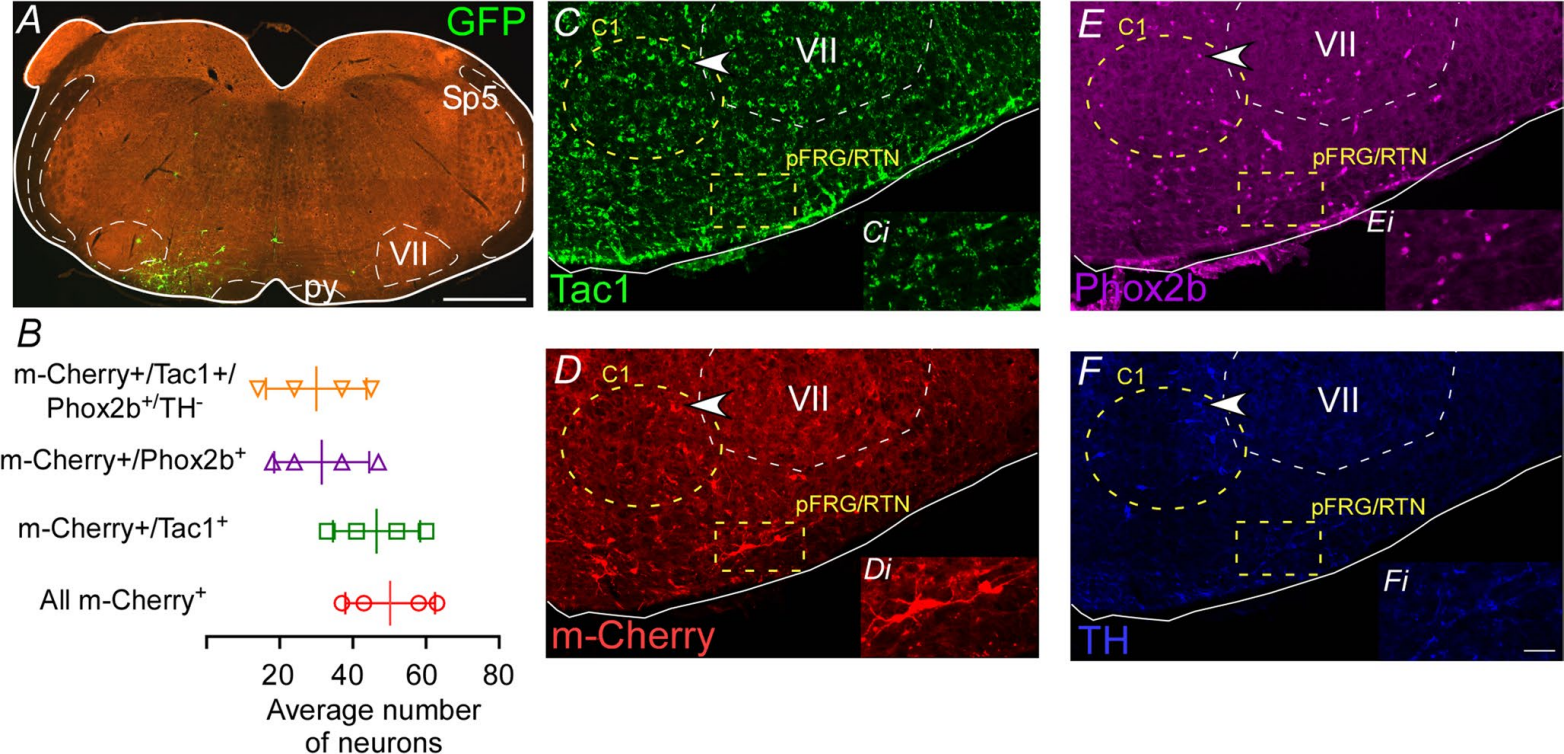
Anatomical distribution of the ChR2-expressing respiratory parafacial neurons. (**A**) Photomicrography showing the injection site of the AAV-EF1a-double-floxed-hChR2(H134R)-mCherry-WPRE-HGHpA into the pFRG/RTN region. (**B**) Cell counts were performed on 1:4 sections at the level of the pFRG/RTM region to quantify the average number of mCherry-positive (red, circle), the double-labeled mCherry and Tac1 (mCherry^+^/Tac1^+^, green, square), the double-labeled mCherry and Phox2b (mCherry^+^/Phox2b^+^, triangle), and the labeled mCherry, Phox2b and Tac1 that is not TH (mCherry^+^/Tac1+/Phox2b^+^/TH^−^, inverted triangle). C-F) Photomicrography showing the immunohistochemistry for (**C**) Tac-1-expressing cells (Tac1^cre/+^::Ai6 mice), (**D**) mCherry (Alexa 647, red), (**E**) Phox2b (Alexa 594, magenta, F) TH (DyLight, blue). Ci-Fi) Represents a higher magnification of the yellow square depicted in C-F. Abbreviations: py, pyramid tract; Sp5, spinal trigeminal nucleus; VII, facial motor nucleus. *N* = 4/group of mice). Scale bar = 1 mm in A; 20 μm in Fi applied to Ci-Fi

**Fig. 5:**
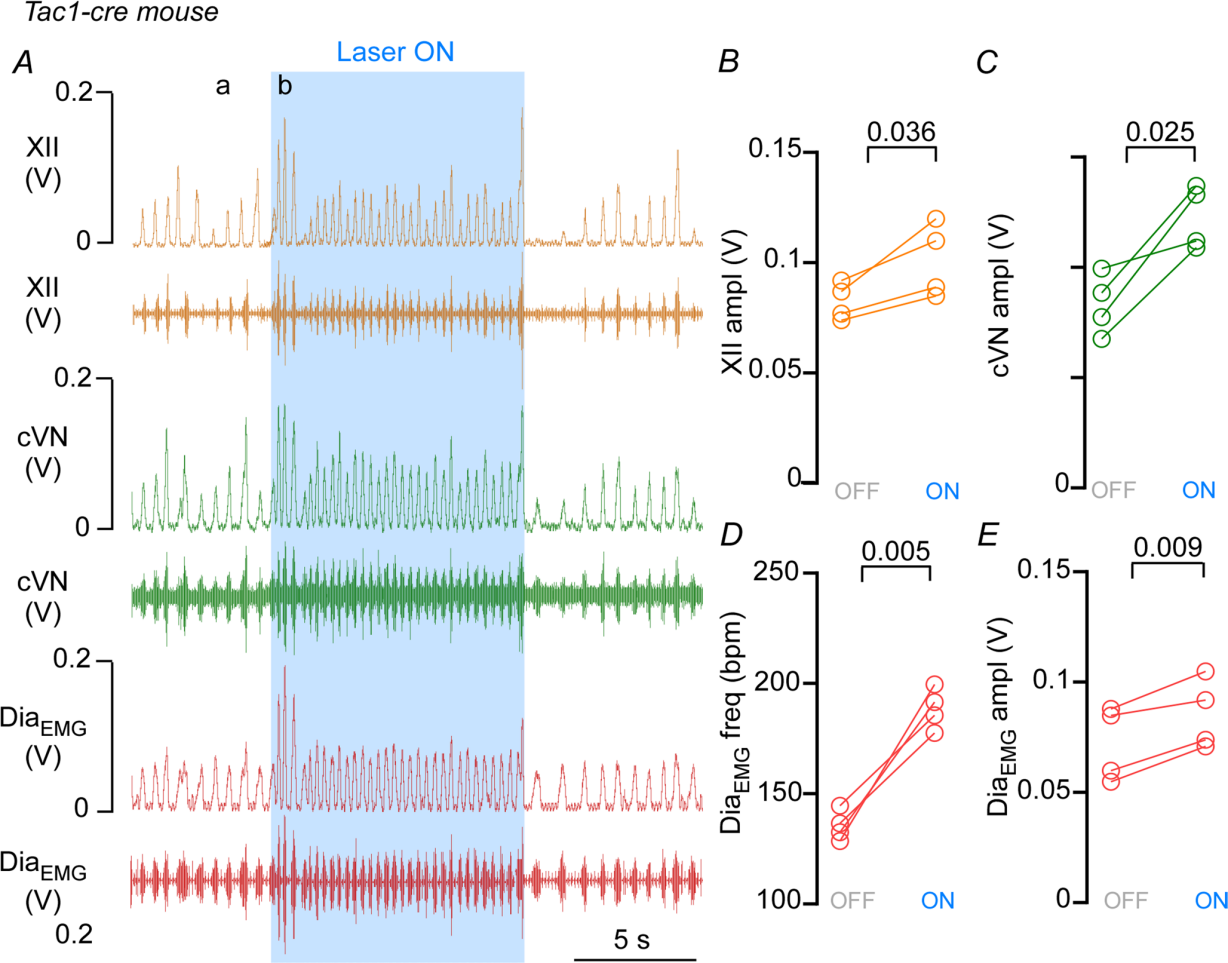
Selective activation of Tac-1 neurons in the ventral aspect of the respiratory parafacial region increases breathing activity in anesthetized mice. **A**) Representative recordings of hypoglossal (XII), cervical vagus nerve (cVN), and diaphragm (Dia_EMG_) activities from an anesthetized Tac-1^Cre/+^::Ai6 mouse, transfected with AAV1-EF1a-double-floxed-hChR2(H134R)-mCherry-WPRE-HGHpA into the pFRG/RTN, under (**a**) baseline and (**b**) during photostimulation (15 Hz, 10 ms light pulse). **B**-**E**) Group data (*N* = 4 mice of the Tac-1^Cre/+^::Ai6 group) showing the change in **B**) XII amplitude (paired t-test, t = 3.63; *p* = 0.036), **C**) cVN amplitude (paired t-test, t = 4.19; *p* = 0.025), **D**) Dia_EMG_ frequency (paired t-test, t = 7.21; *p* = 0.005), **E**) Dia_EMG_ amplitude (paired t-test, t = 5.98; *p* = 0.009) elicited by optogenetic stimulation of Tac-1 neurons in the pFRG/RTN region

 Pharmacological manipulation of NK1 receptors in the pFRG/RTN has been shown to increase both breathing frequency and amplitude (Figs. 1B and 2E-G in the [6]). However, the selective activation of tachykinin-expressing neurons within the pFRG/RTN has not yet been investigated. To explore the potential role of a specific neuronal population in the ventral pFRG, we focused on neurons expressing Tac1, the gene encoding the precursor of substance P. These peptides are strongly expressed in subsets of neurons primarily localized to the ventral pFRG [48, 52] To examine the roles of this peptidergic populations for breathing, Tac1^Cre/+^ mice received unilateral injections of AAV1-EF1a-double-floxed-hChR2(H134R)-mCherry-WPRE-HGHpA into the ventral aspect of the pFRG to allow specific activation of Tac1 neurons with light via optical fibers inserted above the pFRG/RTN region (Fig. 4 A) The histological experiments were designed to verify whether in Tac1^Cre/+^ mice, there is selective transfection of Phox2b-expressing neurons. In Tac1^Cre/+^::Ai6 mice, 65 ± 9.3% of transduced neurons had a Phox2b-immunoreactive nucleus within the ventral aspect of the pFRG region (counted in 1-in-4 sections; *N* = 4) (30 ± 13.7, vs. mCherry^+^/Tac1^+^: 46.5 ± 11.9 neurons) (Figs. 4B) During photoactivation (10-ms pulses at 15 Hz for 10 s) of Tac1 neurons in the pFRG/RTN region, an immediate and sustained change in breathing output was elicited (Fig. 5 A). Indeed, photostimulation of Tac1 pFRG neurons led to an increase in breathing frequency (Dia_EMG_ freq: 188 ± 9.3 vs. baseline: 135 ± 6.8 breaths/min; t = 7.21, df = 3, *p* = 0.005) and Dia_EMG_ amplitude (t = 5.98, df = 3, *p* = 0.009) (Fig. 5 A and D-E). Tac1 neurons stimulation was able to increase both hypoglossal nerve (XII ampl: t = 3.646, df = 3, *p* = 0.0356) and vagus nerve amplitude (cVN ampl: t = 4.19, df = 3, *p* = 0.0248) (Fig. 5 A and B-C).

## Discussion

 The present study provides converging pharmacological, anatomical, and optogenetic evidence that tachykinergic signaling within the ventral aspect of the pFRG plays a critical role in the modulation of respiratory output under both basal and hypercapnic conditions. Specifically, we demonstrate that local activation of substance P signaling within the ventral pFRG increases respiratory drive, that this effect is mediated by NK1 receptors, and that blockade of these receptors attenuates the ventilatory response to hypercapnia. Furthermore, we identify a predominantly glutamatergic population of NK1-R expressing neurons in this region and show that optogenetic activation of Tac1-expressing neurons elicits coordinated activation of respiratory outputs.

### Tachykinergic modulation of baseline respiratory activity

 Microinjection of substance P into the ventral pFRG significantly enhances respiratory activity which is reflected by an increased respiratory frequency, amplitude, and minute volume. This excitatory effect was completely abolished by prior NK1 receptor blockade, demonstrating that SP acts via NK1-R in this region to modulate respiratory output. Importantly, control injections placed outside the pFRG, including within the facial motor nucleus or its dorsal aspect, failed to produce respiratory effects, confirming the functional specificity of the ventral pFRG in mediating tachykinergic excitation. These results are in agreement with previous work demonstrating dense NK1-R expression in the ventral lateral medulla [40, 55, 60, 66] and expand upon them by identifying a functionally relevant SP-sensitive region within the pFRG/RTN region capable of influencing baseline breathing.

### NK1 receptors contribute to hypercapnic chemoreflex responses

 Beyond basal modulation, our study demonstrates that NK1 receptor signaling in the ventral pFRG is required for the full expression of the hypercapnia-induced ventilatory response. Bilateral blockade of NK1 receptors significantly blunted the typical increases in respiratory frequency, amplitude, and minute volume elicited by 7% inspired CO_2_. This finding suggests that tachykininergic transmission in the pFRG/RTN contributes to CO_2_ chemoreception or the downstream relay of chemosensory signals to respiratory motor circuits. Given the proximity of this region to chemosensitive RTN neurons, it is plausible that NK1-R-expressing neurons either represent a chemosensitive subpopulation or form part of a broader circuit that amplifies or integrates central and peripheral chemoreceptive inputs. Consistent with this idea, our data demonstrates the co-localization of neurons expressing Tac1- the gene encoding the precursor of substance P- with Phox2b, a marker of chemosensitive neurons in the RTN. This finding is also consistent with the study by Yeh and colleagues [66] Although we have not yet explored the full spectrum of tachykinin receptors, our findings suggest that the receptor mediating the observed effects belongs to the class of metabotropic tachykinin receptors coupled to membrane-bound G proteins. Specifically, NK1 receptors, which are activated by substance P, appear to modulate neuronal activity within both the pFRG - as demonstrated by our data - and the preBötC, as previously reported [16, 27, 47, 53, 61, 62, 64]. Activation of NK1-R is thought to enhance neuronal excitability by engaging a non-selective cation conductance, likely mediated by a leak sodium (Na⁺) current. Neuronal excitability results from a finely tuned balance between inward (depolarizing) and outward (hyperpolarizing) currents. The RTN neurons possess an intrinsic chemosensitivity that is attributable, at least in part, to a pH-sensitive background K^+^ current [25, 37, 63]. Depolarizing currents, in turn, are thought to depend on voltage-gated Na⁺ channels, a mechanism well established in the preBötC [24, 54]. A strong candidate for such depolarizing current is the NALCN (Na⁺ leak channel, non-selective), which has been implicated in baseline neuronal excitability [10, 28], and which is essential for survival [66] Although structurally related to voltage-gated Na⁺ channels, NALCN is tetrodotoxin-resistant and forms a voltage-insensitive, non-selective cation channel that primarily conducts Na⁺ under physiological conditions. Importantly, NALCN activity can be upregulated by several neuromodulators, including substance P [29]. Genetic deletion of NALCN in mice causes severe respiratory depression and postnatal lethality [28, 66], and NALCN mutations in humans are associated with respiratory dysfunction [3, 10, 14]. These observations point to a mechanistic link between NK1 receptor signaling and NALCN channel function, a relationship we intend to further explore in future studies Indeed, previous work has shown that NALCN mediates the excitatory effects of SP on pFRG/RTN neurons: in mice lacking NALCN, SP-induced increases in neuronal firing were nearly abolished [49]. Although NK1 and 5-HT2 receptors are co-expressed in many pFRG/RTN neurons and are both coupled to Gq-PLC signaling pathways, serotonin (5-HT) continues to exert its excitatory effects even in the absence of NALCN, indicating that SP and 5-HT engage distinct intracellular signaling mechanisms [49] Recent studies suggest that 5-HT acts through 5-HT2 and 5-HT7 receptors to modulate KCNQ and HCN channels via the PLC-PKC and cAMP pathways, respectively [19, 20]. In contrast, SP-mediated responses in pFRG/RTN neurons appear to be independent of KCNQ channels [20]. These findings support the hypothesis that NK1 receptors may activate NALCN through a G protein–independent mechanism, involving intracellular partners such as UNC-80 and Src family kinases, as previously demonstrated in other neuronal populations [28].

### Cellular identity of NK1 receptor-expressing neurons

 Using genetic reporter lines and immunohistochemistry, we further characterized the phenotype of NK1-R expressing neurons in the ventral pFRG. Our analysis revealed substantial co-localization with VGlut_2_-expressing neurons, suggesting a predominantly excitatory glutamatergic phenotype. In contrast, colocalization with VGat-positive (GABAergic) neurons were sparse. These findings suggest that NK1-R-expressing neurons in the ventral pFRG likely act as excitatory interneurons or output neurons that drive respiratory-related motor activity. This is consistent with their role in facilitating inspiratory rhythmogenesis and chemosensory responsiveness as proposed by Yeh and colleagues [66]. Importantly, our study suggests that the Tac1 neurons in the pFRG region play a critical role, not only in chemosensation, but also rhythmogenesis. This raises the questions to what extent Tac1 neurons form a contiguous column with the preBötC, and whether rhythmogenesis is a function that is distributed along the entire VRC by neurons that are modulated by and release substance P. The concept of a network of rhythmogenic neurons that is distributed along the entire VRC which includes the preBötC, but also regions caudal and rostral of the preBötC including the pFRG is also consistent with optogenetic stimulations, lesion experiments and Neuropixel recordings [5, 8].

### Optogenetic activation of Tac1-expressing neurons within the pFRG region evokes respiratory responses

 To directly assess the functional role of tachykinin-producing neurons in the pFRG/RTN region, we selectively activated Tac1-expressing neurons using optogenetics. After transfection the pFRG/RTN region, the ChR2-mCherry fusion protein was detected overwhelmingly in Phox2b-expressing neurons (65%). Most transfected neurons were non-catecholaminergic and non-cholinergic and therefore belonged to a cell group that is strongly activated by hypercapnia in vivo and by acidification in vitro [1, 37, 43, 56] Photostimulation of these neurons elicited a rapid and robust increase in respiratory frequency, accompanied by enhanced activity in the hypoglossal and vagal motor outputs, indicating that Tac1 neurons exert an excitatory influence on respiratory premotor and motor networks. This finding supports the emerging view that the ventral pFRG is a key integrative hub for respiratory activities, where distinct populations of excitatory neurons coordinate breathing with other visceral functions [33, 57, 59] Taken together, these findings provide strong evidence that tachykininergic signaling in the pFRG plays an important role in regulating and modulating breathing outputs. This integrative role of Tac1 neurons reinforces the concept that respiratory outputs are functionally coupled via specific brainstem microcircuits, a mechanism likely essential for maintaining homeostasis during physiological challenges such as hypoxia, hypercapnia, or stress.

### Functional implications and future directions

 Together, these findings establish that tachykinergic signaling within the ventral pFRG is a potent modulator of respiratory rhythm and a key contributor to the ventilatory response to hypercapnia. The predominance of excitatory glutamatergic NK1-R-expressing neurons in this region, along with their capacity to coordinate respiratory outputs, underscores their potential importance in respiratory homeostasis and autonomic regulation. From a translational perspective, these mechanisms may be relevant in pathophysiological states such as congenital central hypoventilation syndrome (CCHS), where central chemosensory pathways are impaired. Enhancing tachykinergic signaling or selectively targeting Tac1 neurons in the pFRG could represent a novel strategy for restoring respiratory function in such conditions Future studies should examine the developmental origin, upstream inputs, and downstream targets of Tac1/NK1-R-expressing neurons in the pFRG/RTN to fully elucidate their role in the respiratory network. Moreover, investigating their interaction with other neuromodulatory systems and their potential plasticity under conditions of chronic hypoventilation or disease may offer insights into compensatory mechanisms within the respiratory control network.

## Data Availability

No datasets were generated or analysed during the current study.
